# Validity of Smartphone-Based Measurement for Assessing Lower Limb Power for Sarcopenia and Frailty Discrimination: Cross-Sectional Study

**DOI:** 10.2196/83805

**Published:** 2026-04-06

**Authors:** Shuichi P Obuchi, Hisashi Kawai, Takeshi Kera, Keigo Imamura, Manami Ejiri, Hirohiko Hirano, Kazushige Ihara, Hiroyuki Sasai, Yoshinori Fujiwara

**Affiliations:** 1 Tokyo Metropolitan Institute for Geriatrics and Gerontology, 35-2 Sakae-cho, Itabashi-ku, Tokyo, 1730015, Japan, 81 3-3964-3241 ext 4243, 81 3-3964-1844; 2 Department of Physical Therapy, Takasaki University of Health and Welfare, Takasaki, Japan; 3 Faculty of Medicine, Hirosaki University, Hirosaki, Japan

**Keywords:** acceleration, aging, frailty, sarcopenia, smartphone

## Abstract

**Background:**

Increasing life expectancy has increased focus on the health-related consequences of aging, such as sarcopenia and frailty. Given the prevalence of these conditions among older individuals and the frequent resulting long-term care needs, early detection and intervention are crucial.

**Objective:**

This study aimed to validate a novel smartphone-based system measuring acceleration during the sit-to-stand movement to detect sarcopenia and frailty.

**Methods:**

Participants were 587 individuals from the Otassha study cohort who underwent health assessments in 2023, of whom 569 (96.9%) completed 2 supervised sit-to-stand trials while holding a smartphone on the lower abdomen. Sarcopenia and frailty were diagnosed using the Asian Working Group for Sarcopenia 2019 criteria and the revised Japanese Cardiovascular Health Study criteria, respectively. Peak force, rising time (T1), and stabilization time (T2) were extracted from acceleration signals, and reproducibility was examined using the intraclass correlation coefficient (ICC(2,1)). Predictive models were developed using elastic net penalized logistic regression, and model performance was evaluated using 500 bootstrap resamples. Benchmark models using age and sex, walking speed, and grip strength were also constructed for comparison.

**Results:**

Sarcopenia and frailty were identified in 16.7% (95/569) and 9% (51/569) of the participants, respectively. Peak force demonstrated excellent reliability (ICC=0.863), whereas T1 and T2 showed lower reproducibility (ICC<0.30). For sarcopenia, the smartphone model achieved a bootstrap area under the receiver operating characteristic curve (AUC) of 0.800 and an optimism-corrected AUC of 0.781 (95% CI 0.733‐0.826), outperforming walking speed (0.663) and age and sex (0.656) and ranking second only to grip strength (0.845). For frailty, the smartphone model showed moderate discrimination, with an optimism-corrected AUC of 0.659 (95% CI 0.587‐0.736), exceeding age and sex (0.604), whereas walking speed remained the strongest predictor (0.751).

**Conclusions:**

Smartphone-derived sit-to-stand acceleration provides a practical and scalable approach for screening for sarcopenia and frailty in community-dwelling older adults. While traditional indicators such as grip strength and walking speed remain the most accurate predictors, smartphone-based measurements offer meaningful complementary information and may support large-scale functional screening and early detection initiatives in superaged societies.

## Introduction

As people live longer, health issues shift from being related to diseases to being related to aging [1]. In Japan, the proportion of individuals requiring nursing care increases exponentially with age. Statistics indicate that 48.1% of individuals aged 80 years and older are certified as needing nursing care [2]. Furthermore, in medical institutions, older patients must consider aging alongside their underlying illnesses [3-5]. Therefore, the interest in sarcopenia and frailty has increased [6,7]. Research papers in PubMed have identified that the use of “sarcopenia” and “frailty” as keywords has increased by 103% and 98%, respectively, over the past 5 years. Many studies on sarcopenia and frailty were published in the 1990s, including those on the effects of high-intensity strength training in older people [8]. A consensus was reached that sarcopenia and frailty can be reversed with the appropriate intervention at the right time even though they are phenomena associated with aging [8-10].

Taking countermeasures against sarcopenia and frailty is challenging given the gradual development of the associated symptoms [11,12], which highlights the necessity for triggers that prompt behavior change. A system that can monitor indicators of sarcopenia and frailty, similar to how weight scales and blood pressure monitors are instrumental in managing lifestyle-related diseases, is urgently needed. The diagnostic criteria for sarcopenia and frailty, as outlined by the European Working Group on Sarcopenia in Older People [13] and the Fried frailty index [14], require an initial screening that includes physical performance tests, such as walking speed, and measures of dynapenia, such as grip strength. Although we previously developed a validated system using smartphone GPS data to measure walking speed [15,16], this system does not assess dynapenia. This limitation is significant because dynapenia is a crucial factor in the comprehensive diagnosis of sarcopenia and frailty. Moreover, the definition of frailty proposed by Howlett et al [6] as an accumulation of deficits underscores the importance of a multifactorial evaluation of frailty. This further indicates the necessity of expanding our system’s capabilities to include a wider range of diagnostic criteria.

The sit-to-stand test, reportedly a simple way to evaluate lower limb power [11], is considered a useful standard for determining frailty [12], risk of falling [17], and risk of disability [18] and can be undertaken on a daily basis. The only issue is that a skilled tester is required to perform the measurements [19-21]. Our research investigated the potential of using ground reaction force (GRF), captured via standard weight scales during standing, as a novel metric for assessing the strength of the lower limb muscles and detecting indications of sarcopenia [22,23]. We found that the discriminant for sarcopenia probability could be ascertained from only the peak vertical GRF during the sit-to-stand movement, with a sensitivity of 89.7% and specificity of 80.5%. Because force is the product of mass and acceleration according to Newton’s second law, measuring an object’s acceleration allows one to estimate force anywhere without relying on the reaction force measurement. Thus, the validity of estimating GRF using an accelerometer has been widely studied [24-26]. Because smartphones are equipped with built-in accelerometers [27], they can likely estimate the peak GRF during the sit-to-stand movement. Several previous studies have reported that smartphone accelerometers can measure human movement with accuracy comparable to that of laboratory-grade accelerometers [28,29].

We considered that, if smartphones could detect sarcopenia from acceleration measurements without measuring GRF, early diagnosis of sarcopenia and frailty would be possible even at home, facilitating early recognition of functional decline and flagging the need for behavior changes. Thus, in this study, we used acceleration measured using a smartphone during the sit-to-stand movement to determine whether sarcopenia could be discerned without requiring GRF measurement.

## Methods

### Study Design

This was a cross-sectional study.

### Participants

We included 587 people from the cohort of the Otassha study, a longitudinal study that has been ongoing since 2011 [30], who underwent an invitation-based health checkup and completed sarcopenia, frailty, and sit-to-stand measurements from September 2023 to October 2023. All study participants lived in the community and were aged over 65 years. Participants living in care facilities, as well as individuals with contraindications for bioelectrical impedance analysis, were excluded from the study. Participants underwent comprehensive geriatric health examinations regarding their physical, cognitive, psychological, nutritional, and lifestyle habits.

### Body Composition and Physical Function Measurements

Body composition was measured using a bioimpedance body composition meter (InBody 770; InBody Co, Ltd) to estimate appendicular skeletal muscle mass. Although dual-energy x-ray absorptiometry is recognized as the gold standard for measuring body composition, bioimpedance analysis has been evaluated against it and verified to provide correct measurements [31].

The participants were instructed to stand still with their arms at their sides while using the bioimpedance equipment without talking and to hold a sensor for measurement. The skeletal muscle index (SMI) was calculated as the appendicular skeletal mass, estimated from the body composition measurement, divided by the square of the individual’s height. Smedley hand dynamometers were used to measure grip strength.

For grip strength measurements, participants were asked to extend their arms straight down and grip the dynamometer as strongly as possible with their preferred hand when the tester instructed them to do so.

Walking speed was measured on a 5-m course with a preceding 3-m acceleration and subsequent 3-m deceleration walkway. Participants were asked to walk the course once at a comfortable speed. The time taken to walk through the 5-m section of the course was measured using a digital stopwatch.

### Acceleration Measurement Using a Smartphone During Sit-to-Stand Movement and Force Estimation

**Figure 1.**
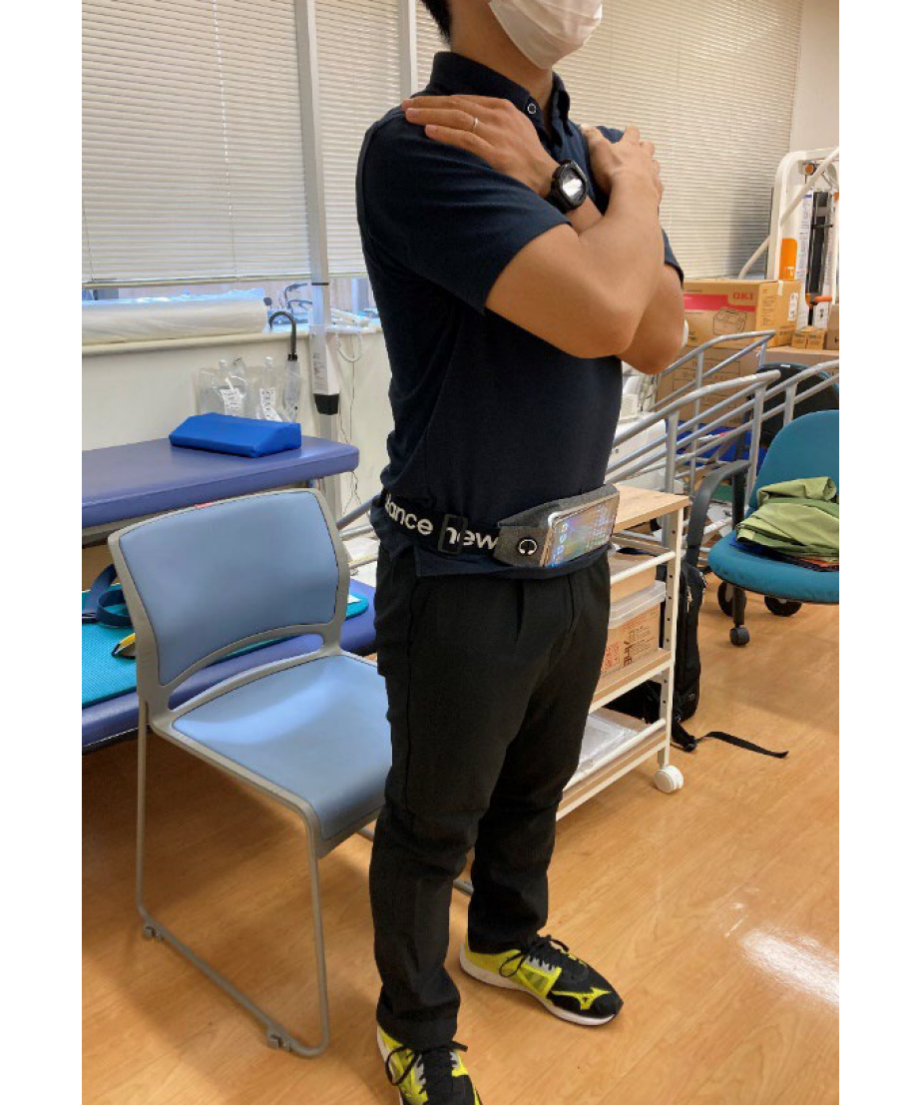
Smartphone configuration.

A smartphone with our app installed was secured to the lower abdomen of each participant using a commercially available sports smartphone holder (Figure 1). Participants folded their arms across their chests to minimize arm motion and trunk variability. Participants received auditory prompts from a Bluetooth speaker positioned 2 m in front of them via the smartphone app, which instructed them to stand up as quickly as possible and then hold the position for 3 seconds. After the practice attempt, the participants were evaluated twice, transitioning rapidly from a seated resting position (seat height; 42 cm) to a standing position. Acceleration during this action was captured using the smartphone app.

The app is web based and uses JavaScript to call the operating system application programming interface and measure the smartphone’s acceleration on 3 axes over a given period. To validate the accuracy of the smartphone accelerometer for use in biomechanical research, 3 healthy volunteers performed sit-to-stand trials at 3 different speeds (fast, moderately fast, and normal) to simulate a range of physical performance levels (9 trials in total). A research-grade reference accelerometer (MVP-RF8-BC; MicroStone Corp; sampling at 1000 Hz) was attached to the smartphone holder on the participants’ lower abdomen to measure acceleration simultaneously with the smartphone. The resultant acceleration signals from both devices were synchronized using cross-correlation to align the waveforms temporally. We then extracted the specific “movement phase” (from the start of the rise to stabilization) and assessed the waveform similarity using the intraclass correlation coefficient (ICC(2,1); absolute agreement). The analysis demonstrated excellent agreement between the smartphone and the reference device despite the difference in sampling rates. The mean ICC(2,1) was 0.982 (SD 0.019), with a range of 0.932 to 0.995 across all trials and speeds, confirming that the smartphone’s accelerometer measurements were reliably comparable to those of the dedicated biomechanical research device.

The smartphone app recorded the sit-to-stand motion at 60 Hz, and this motion was analyzed in the cloud. Data were processed using an inverse fast Fourier transform algorithm with a low-pass filter at 7.5 Hz to mitigate the impact of noise outside the physical activity domain. In traditional settings, the maximum force exerted during sit-to-stand motion is measured using a floor reaction force meter with a fixed coordinate system established upon installation. However, when using a smartphone accelerometer attached to the body, the coordinate system is affected by the user’s movements. The norm of the triaxial acceleration was computed to account for these variations and ensure that the measurement was less affected by changes in the orientation of the accelerometer. The peak force exerted during the sit-to-stand motion was calculated by multiplying the resultant peak acceleration by the body weight.

### Measurement of Peak Acceleration and Duration of Movement in the Sit-to-Stand Transition

**Figure 2.**
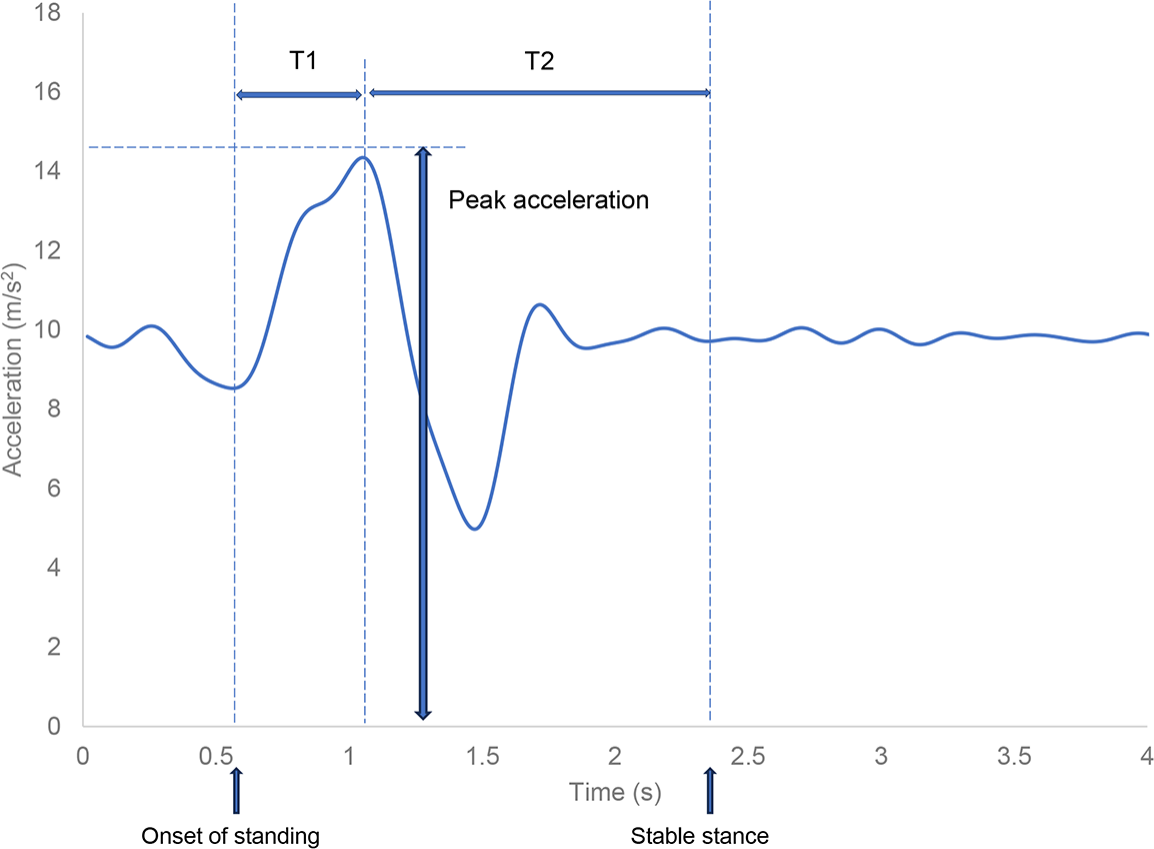
Definition of measurements.

The onset of standing was identified as the point at which the accelerometer registered the lowest acceleration from the start of the motion (Figure 2). Peak acceleration (measured in m/s^2^) was defined as the highest acceleration recorded after starting. A stable stance was considered when the acceleration variation decreased to 1% or less of the gravitational acceleration after peak acceleration. The duration from the start to peak acceleration was termed the “rising time” (T1; seconds), whereas the duration from peak acceleration to attainment of a stable stance was termed the “stability time” (T2; seconds). The peak force (in newtons) was calculated by multiplying peak acceleration by the weight of each participant.

### Definition of Sarcopenia

Sarcopenia was diagnosed based on the criteria outlined in the 2019 consensus statement of the Asian Working Group for Sarcopenia [32]. Grip strength below 28 kg for men and below 18 kg for women, walking speed below 1.0 m/s, and SMI below 7.0 kg/m^2^ for men and below 5.7 kg/m^2^ for women were the thresholds used to diagnose sarcopenia. Participants who met the criteria for SMI and grip strength or walking speed were classified as having sarcopenia. However, sarcopenia severity was not classified, similar to our previous study [22].

### Definition of Frailty

Frailty was diagnosed using the criteria outlined in the Japanese version of the Cardiovascular Health Study [14]. The criteria were an adaptation of the original Fried frailty index [33] tailored to the Japanese population. A person was classified as frail if they met 3 or more of the following criteria: an unintentional loss of 2 kg or more of body weight in the previous 6 months, handgrip strength of less than 28 kg for men and less than 18 kg for women, a feeling of exhaustion, a walking speed slower than 1.0 m/s, and a lack of engagement with regular physical activity.

### Statistical Analysis

To confirm the reproducibility of the smartphone-derived measurements (peak force, T1, and T2), we calculated the ICC(2,1) using a 2-way mixed-effects, absolute-agreement model based on 2 consecutive sit-to-stand trials. For all subsequent analyses, the average of the 2 measurements was used.

To identify and classify sarcopenia and frailty, we developed predictive models following TRIPOD (Transparent Reporting of a Multivariable Prediction Model for Individual Prognosis or Diagnosis) recommendations. Five logistic regression models were constructed for each outcome to enable direct comparison with established clinical indicators:

- Age and sex model
- Walking speed model
- Grip strength model
- Smartphone model, incorporating age, sex, peak acceleration–derived force, rising time (T1), and stabilization time (T2)
- Smartphone reduced model, incorporating age, sex, and derived force

All models were implemented as elastic net penalized logistic regression (penalty=“elasticnet”; l1_ratio=0.5; solver=saga) to mitigate overfitting and address multicollinearity among biomechanical and functional predictors. Continuous predictors were standardized using *z*-score transformation.

Internal validation was conducted using bootstrap resampling (500 iterations). For each model, we calculated the following performance metrics: apparent area under the receiver operating characteristic curve (AUC), bootstrap AUC (mean), bootstrap 95% CI, optimism-corrected AUC, corrected AUC 95% CI, calibration slope, calibration intercept, sensitivity, and specificity.

Calibration was assessed using a logistic recalibration model, from which the calibration slope and intercept were derived. We reported apparent and bootstrap optimism-corrected calibration metrics (500 resamples; percentile 95% CIs). Calibration slopes close to 1.0 and intercepts near 0 indicated good agreement between predicted and observed risk.

To contextualize the performance of the smartphone-based model, we additionally compared it against established screening measures—walking speed, grip strength, and age and sex—using identical model structures and validation procedures.

All statistical analyses and model development were conducted using Python (version 3.11; Python Software Foundation), including scikit-learn (Google Summer of Code project; logistic regression and scaling), NumPy, and pandas. The reproducibility analysis (ICC) and descriptive statistics were performed and calculated using SPSS (version 27; IBM Corp).

### Ethical Considerations

This study was conducted as part of the aforementioned comprehensive cohort study. The research plan was discussed and approved by the ethics committee of the Tokyo Metropolitan Institute for Geriatrics and Gerontology (R22-034). Written informed consent was obtained from all participants. Strict confidentiality procedures were implemented. All analyses were performed using only anonymized datasets. Participants did not receive any incentives.

## Results

### Participant Flow

**Figure 3.**
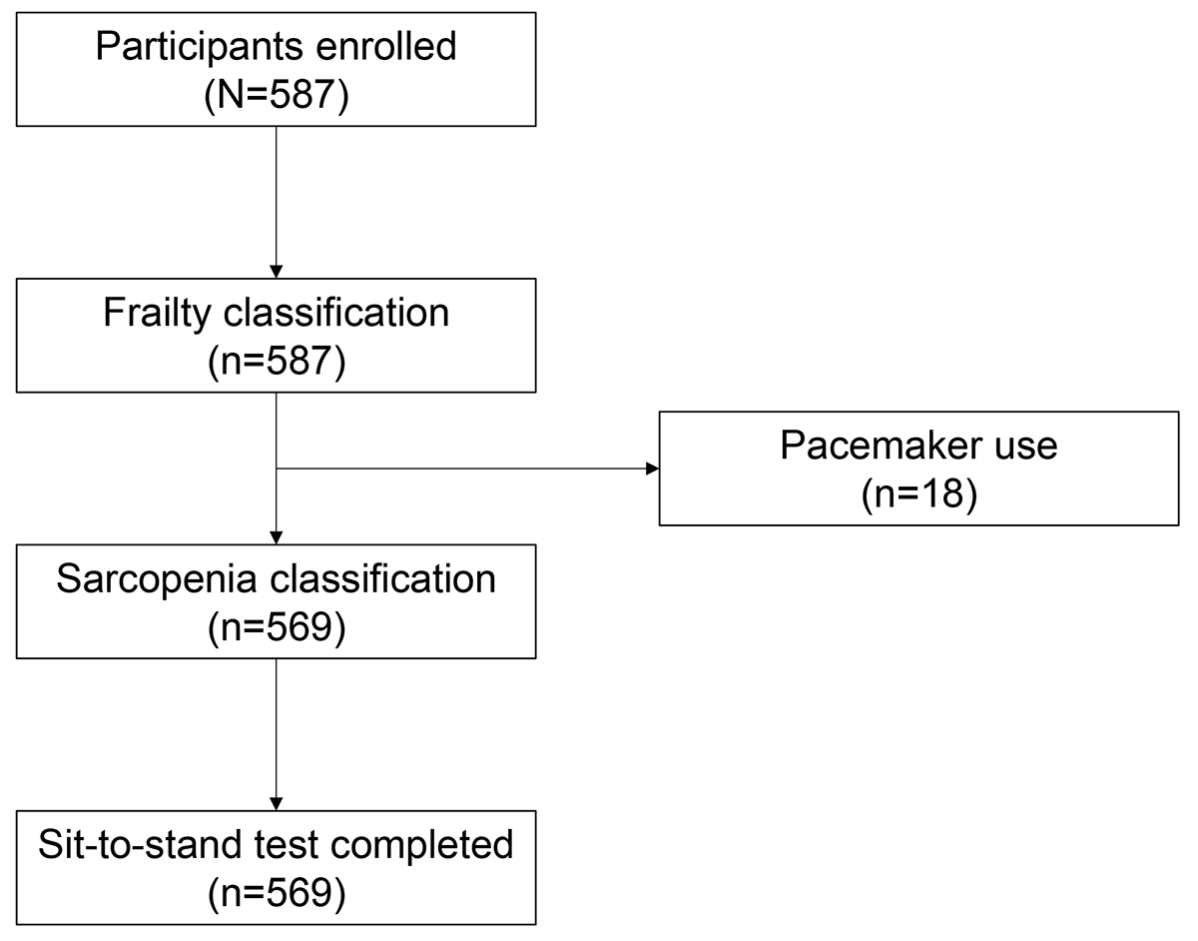
Participant flow diagram.

A total of 587 older adults participated in the assessment, of whom 569 (96.9%) completed the smartphone-based sit-to-stand measurement (Figure 3).

### Participant Characteristics

The participants’ characteristics are listed in Table 1. Among them, 95 participants (n=25, 26.3% men and n=70, 73.7% women) met the criteria for sarcopenia, and 51 participants (n=22, 43.1% men and n=29, 56.9% women) met the criteria for frailty. Compared with individuals without sarcopenia, those with sarcopenia were older (mean age 77, SD 7 years vs 73, SD 6 years; *P*<.001) and shorter and had less weight, weaker grip strength (mean 16, SD 5 kgf vs 26, SD 8 kgf; *P*<.001), slower walking speed (mean 1.25, SD 0.22 m/s vs 1.40, SD 0.23 m/s; *P*<.001), and lower peak force (mean 70.87, SD 14.98 N vs 92.56, SD 25.20 N; *P*<.001). Similarly, individuals with frailty were older (mean age 77, SD 8 years vs 73, SD 6 years; *P*=.003) and had lower grip strength (mean 19, SD 6 kgf vs 24, SD 8 kgf; *P*<.001), slower walking speed (mean 1.13, SD 0.25 m/s vs 1.40, SD 0.23 m/s; *P*<.001), and lower peak force (mean 78.56, SD 20.49 N vs 89.93, SD 25.25 N; *P*<.001) than individuals without frailty (Table 2).

**Table 1.** Participant characteristics.

	Male participants (n=223), mean (SD)	Female participants (n=364), mean (SD)
Age (years)	74 (6)	74 (7)
Height (cm)	165.9 (6.6)	152.8 (5.9)
Weight (kg)	65.1 (10.6)	51.6 (8.7)
SBP^a^ (mm Hg)	131 (19)	128 (17)
DBP^b^ (mm Hg)	78 (12)	74 (11)
Grip strength (kgf)	31 (6)	20 (5)

a SBP: systolic blood pressure.

b DBP: diastolic blood pressure.

**Table 2.** Baseline characteristics and frailty phenotype components by sarcopenia and frailty status.

Variable	Without sarcopenia (n=474), mean (SD)	With sarcopenia (n=95), mean (SD)	*P* value	Nonfrail (n=536), mean (SD)	Frail (n=51), mean (SD)	*P* value
Age (years)	73 (6)	77 (7)	<.001	73 (6)	77 (8)	.003
Height (cm)	158.9 (8.7)	152.3 (7.7)	<.001	158.0 (8.8)	155.3 (9.7)	.42
Weight (kg)	58.6 (11.5)	48.2 (7.3)	<.001	56.9 (11.4)	55.2 (12.7)	.31
Grip strength (kgf)	26 (8)	16 (5)	<.001	24 (8)	19 (6)	<.001
Walking speed (m/s)	1.40 (0.23)	1.25 (0.22)	<.001	1.40 (0.23)	1.13 (0.25)	<.001
Peak force (N)	92.56 (25.20)	70.87 (14.98)	<.001	89.93 (25.25)	78.56 (20.49)	<.001
Appendicular SMI^a^ (kg/m^2^)	6.66 (0.99)	5.49 (0.74)	<.001	6.48 (1.03)	6.29 (1.15)	.26

a SMI: skeletal muscle index.

### Reproducibility of Smartphone-Derived Sit-to-Stand Metrics

The reproducibility analysis based on 2 consecutive sit-to-stand trials demonstrated excellent reliability for peak force (ICC(2,1)=0.863). Rising time (T1; ICC=0.270) and stabilization time (T2; ICC=0.278) showed lower reproducibility. Therefore, the mean of the 2 measurements was used for subsequent analyses.

### Frailty Phenotype Components

The distribution of the 5 frailty phenotype components is shown in Table 3.

**Table 3.** Frailty phenotype components.

Component	Nonfrail (n=536), n (%)	Frail (n=51), n (%)
Low walking speed	12 (2.2)	16 (31.4)
Weight loss	59 (11.0)	33 (64.7)
Exhaustion	127 (23.7)	46 (90.2)
Low activity	77 (14.4)	25 (49.0)
Weakness	133 (24.8)	44 (86.3)

Participants classified as frail exhibited a substantially higher prevalence of each phenotype component compared with individuals without frailty. Specifically, participants with frailty demonstrated markedly higher proportions of slow walking speed (16/51, 31.4% vs 12/536, 2.2%), weight loss (33/51, 64.7% vs 59/536, 11%), exhaustion (46/51, 90.2% vs 127/536, 23.7%), low physical activity (25/51, 49% vs 77/536, 14.4%), and weakness (44/51, 86.3% vs 133/536, 24.8%). These differences are consistent with the Japanese Cardiovascular Health Study frailty phenotype criteria and confirm that the frail group exhibited a substantially higher burden of functional and symptomatic deficits.

### Predictive Performance for Sarcopenia

**Figure 4.**
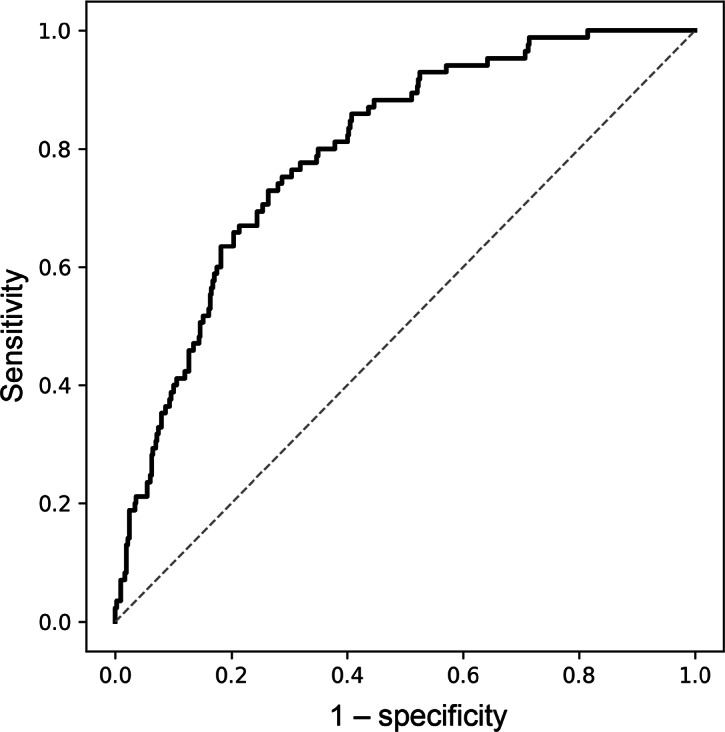
Receiver operating characteristic curve analysis for estimating sarcopenia.

Model performance for sarcopenia is summarized in Table 4. Among the benchmark models, the grip strength model demonstrated the highest discrimination, with a bootstrap AUC of 0.847 (95% CI 0.797‐0.893). The smartphone model (age, sex, peak force, T1, and T2) showed good predictive ability, achieving a bootstrap AUC of 0.800 (95% CI 0.754‐0.848; Figure 4). After internal validation using 500 bootstrap samples, the model achieved an optimism-corrected AUC of 0.781 (95% CI 0.733‐0.826). This performance exceeded that of the walking speed (corrected AUC=0.663) and age and sex (corrected AUC=0.656) models. Calibration of the smartphone model was modest (slope=1.04; intercept=0.06). The results for the smartphone reduced model were similar to those for the full model.

**Table 4.** Performance of models for predicting sarcopenia.

Model	Predictors	AUC^a^, bootstrap mean (95% CI)	Apparent AUC	Optimism-corrected AUC (95% CI)	Calibration slope	Calibration intercept	Sensitivity	Specificity
Age and sex	Age and sex	0.671 (0.599‐0.737)	0.665	0.656 (0.591‐0.725)	1.02	0.04	0.682	0.598
Walking speed	Normal walking speed	0.659 (0.599‐0.719)	0.661	0.663 (0.603‐0.723)	1.02	0.04	0.659	0.614
Grip strength	Grip strength	0.847 (0.797‐0.893)	0.846	0.845 (0.800‐0.895)	1.04	0.05	0.729	0.904
Smartphone	Age, sex, peak force, T1^b^, and T2^c^	0.800 (0.754‐0.848)	0.794	0.781 (0.733‐0.826)	1.04	0.06	0.765	0.689
Smartphone reduced model	Age, sex, and peak force	0.796 (0.752‐0.844)	0.795	0.788 (0.741‐0.832)	1.04	0.05	0.800	0.680

a AUC: area under the receiver operating characteristic curve.

b T1: rising time.

c T2: stability time.

### Predictive Performance for Frailty

Model performance for frailty is shown in Table 5.

**Table 5.** Performance of the model for predicting frailty.

Model	Predictors	AUC^a^, bootstrap mean (SD; 95% CI)	Apparent AUC	Optimism-corrected AUC (95% CI)	Calibration slope	Calibration intercept	Sensitivity	Specificity
Age+sex	Age and sex	0.637 (0.543‐0.727)	0.625	0.604 (0.517‐0.688)	1.05	0.11	0.714	0.486
Walking speed	Normal walking speed	0.750 (0.672‐0.826)	0.750	0.751 (0.674‐0.828)	1.03	0.07	0.857	0.529
Grip strength	Grip strength	0.704 (0.615‐0.781)	0.703	0.703 (0.626‐0.792)	1.05	0.1	0.548	0.829
Smartphone	Age, sex, peak force, T1^b^, and T2^c^	0.720 (0.640‐0.796)	0.697	0.659 (0.587‐0.736)	1.09	0.19	0.476	0.859
Smartphone reduced model	Age, sex, and peak force	0.688 (0.602‐0.768)	0.671	0.649 (0.568‐0.736)	1.07	0.17	0.357	0.889

a AUC: area under the receiver operating characteristic curve.

b T1: rising time.

c T2: stability time.

**Figure 5.**
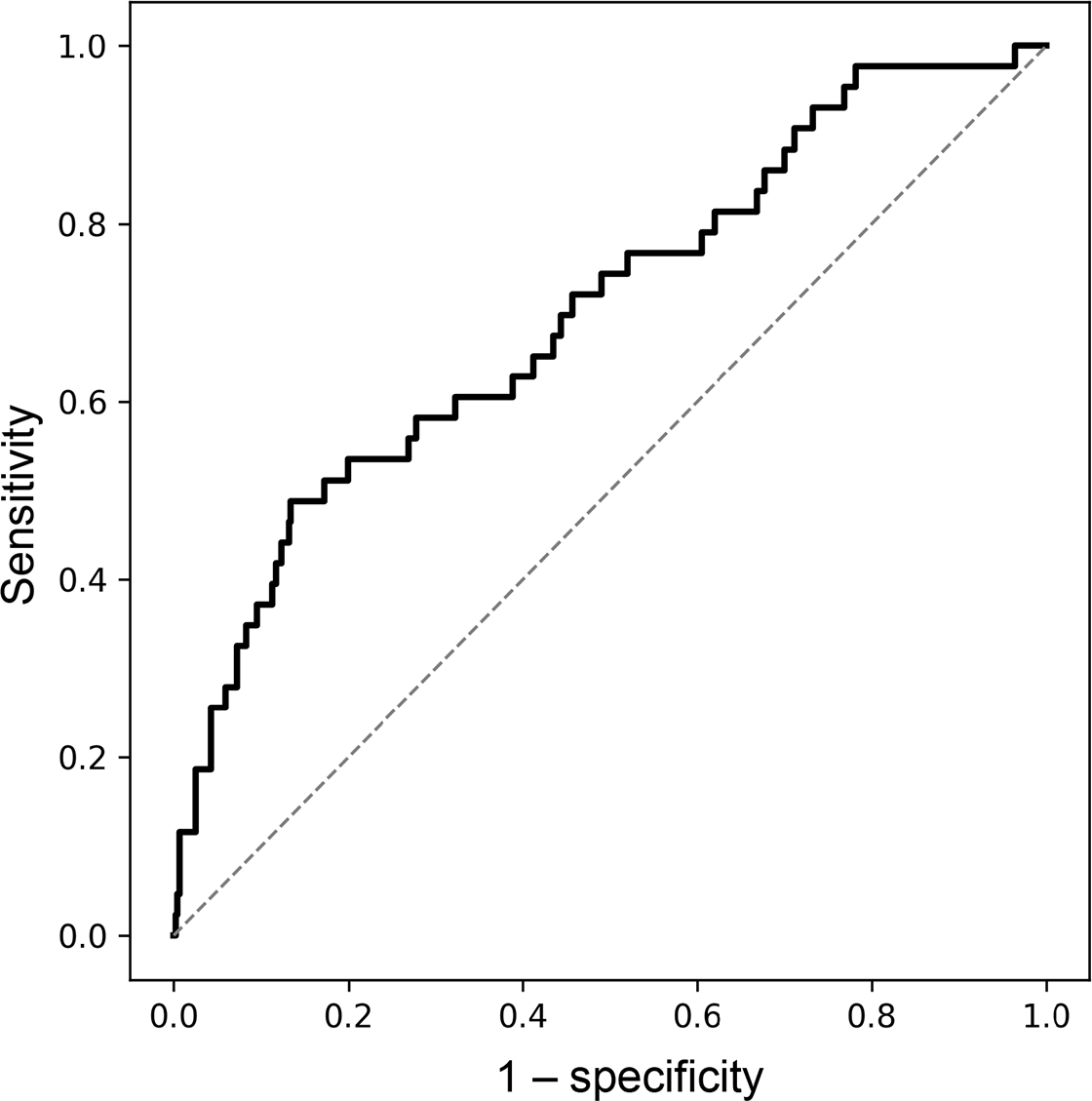
Receiver operating characteristic curve analysis for estimating frailty.

Walking speed demonstrated the highest discrimination (AUC=0.750, 95% CI 0.672‐0.826), followed by grip strength (AUC=0.704, 95% CI 0.615‐0.781). The smartphone model achieved a bootstrap AUC of 0.720 (95% CI 0.640‐0.796; Figure 5). The optimism-corrected AUC was 0.659 (95% CI 0.587‐0.736). This indicates moderate discrimination, outperforming the age and sex model (corrected AUC=0.604). Calibration of the smartphone model remained modest (slope=1.09; intercept=0.19). The smartphone reduced model had a slightly lower AUC than the full model.

### Overall Model Comparison

Across outcomes, the smartphone-derived sit-to-stand measurements consistently outperformed demographic models and walking speed for sarcopenia and provided moderate predictive value for frailty. Grip strength and walking speed remained the strongest predictors of sarcopenia and frailty, respectively, whereas the smartphone model functioned as a strong secondary predictor of sarcopenia and a useful complementary indicator of frailty.

## Discussion

This study investigated the ability of a smartphone’s built-in accelerometer to quantify sit-to-stand motion and estimate the likelihood of sarcopenia and frailty. Using elastic net logistic regression with bootstrap internal validation, we found that smartphone-derived measurements provided clinically meaningful information related to lower limb function. The optimism-corrected AUC values were 0.781 for sarcopenia and 0.659 for frailty, indicating good and moderate discrimination, respectively.

The higher predictive performance for sarcopenia than for frailty is consistent with the nature of their diagnostic constructs. Sarcopenia is defined using specific physical parameters—loss of muscle mass, weakness, and decreased physical performance—measured using indexes such as skeletal muscle mass, grip strength, and walking speed [7]. Because sit-to-stand acceleration is closely linked to lower limb power, the smartphone model aligned well with these characteristics. In contrast, frailty is a multidimensional syndrome involving physical, psychological, and behavioral components, including weight loss, exhaustion, reduced activity, slow walking speed, and weakness [14]. As a result, acceleration-based measurements alone cannot fully capture its complexity, which accounts for the moderate AUC observed.

Importantly, benchmark comparisons with established indicators demonstrated that the smartphone model performed substantially better than demographic factors alone or walking speed alone in predicting sarcopenia. The age and sex model and the walking speed model showed optimism-corrected AUCs of 0.656 and 0.663, respectively, whereas the smartphone model achieved a markedly higher corrected AUC of 0.781, making it the second-best performer after grip strength (corrected AUC=0.845). This indicates that smartphone-derived sit-to-stand acceleration captures key biomechanical features that are strongly aligned with sarcopenia-related muscle weakness. For frailty, walking speed remained the strongest single predictor (corrected AUC=0.751), followed by grip strength (corrected AUC=0.703). The smartphone model demonstrated moderate discrimination, with a corrected AUC of 0.659, outperforming the age and sex model (corrected AUC=0.604) by a meaningful margin. These findings support the role of smartphone-based measurements as a practical and scalable screening method that offers a performance comparable to that of established functional indicators and that complements—rather than replaces—traditional clinical assessments.

Our previous work using GRF from a weight scale reported higher AUC values (0.906 for men and 0.858 for women) [22]. Conceptually, GRF and accelerometer-derived force estimates should be equivalent if the device is perfectly aligned with the body’s center of gravity (COG). However, the COG is located inside the body and cannot be directly accessed. In this study, we placed the smartphone on the lower abdomen to avoid interference with the chair backrest, which likely introduced deviation from the COG and contributed to the lower predictive performance. Differences in participant characteristics between this and our previous study may also have influenced the results.

Despite these differences, the smartphone model demonstrated good discrimination for sarcopenia. Considering that smartphones can also measure walking speed—which is strongly associated with frailty risk—and can integrate questionnaire data, their potential as multifaceted screening tools is substantial. Kawai et al [34] showed that a 1.0 m/s increase in daily walking speed measured via smartphone reduced the risk of frailty to one-fiftieth of that at slower speeds. In this study, we focused intentionally on the biomechanical aspect of dynapenia without incorporating questionnaire items, and the results suggest that predictive accuracy could be further improved by integrating behavioral and self-reported variables.

Moreover, smartphones allow for the integration of questionnaire data, which is essential for a comprehensive frailty assessment. In this study, we focused on whether smartphone measurements could capture dynapenia, a key component in the evaluation of sarcopenia and frailty, without including questionnaire items in the regression equation. Our findings revealed high and moderate AUC values, suggesting that the accuracy of sarcopenia and frailty screening can be further enhanced by adding these questionnaire items and walking speed to the regression model.

Transitioning from this supervised protocol to a scalable, unsupervised screening tool requires addressing practical challenges regarding feasibility and data reliability. In this study, although participants performed the sit-to-stand movement independently following audio instructions from the smartphone, the device was attached to the lower abdomen by a researcher. Consequently, future verification is necessary to determine whether users can correctly attach the device and obtain accurate measurements in a home setting. Furthermore, the “digital divide” presents a significant challenge for older adults, as is common with the adoption of various digital technologies. Addressing these accessibility issues and ensuring ease of use will be critical considerations for the widespread implementation of this screening tool.

Physical and social interventions are known to be effective in improving sarcopenia and frailty; however, timely behavioral adjustments remain challenging [35,36]. In 2006, Japan shifted its nursing care insurance to focus on prevention by implementing a system to screen all older individuals and offer interventions based on their symptoms through the care management process. Despite these measures, many individuals declined to participate in this initiative. One solution may be to enhance awareness of sarcopenia and frailty as health issues in old age and choose better behaviors from middle age onward when these issues become noticeable. Antonovsky [37] proposed salutogenesis; however, we believe it is important to use our internal power to adopt as many behaviors as possible that are less likely to lead to sarcopenia and frailty rather than relying on outsiders to persuade us to change our behavior. Therefore, understanding the challenges associated with aging from midadulthood is essential. A system such as the one we introduced, which enables measurements anytime and anywhere, could prove to be an effective starting point.

This study has limitations. First, the temporal variables (T1 and T2) showed low reproducibility, indicating a need for improved signal processing. Second, measurements were performed under standardized, supervised conditions with a fixed chair height, which may differ from those obtained in unsupervised real-world environments. Third, the cohort consisted of relatively healthy, community-dwelling older adults, limiting the generalizability of the findings to more frail or institutionalized populations. Finally, external validation using an independent dataset is required to fully establish the robustness of the prediction models.

In conclusion, smartphone-derived sit-to-stand acceleration provides a feasible and scalable method for estimating sarcopenia and frailty risk. Although not a replacement for clinical diagnostic assessments, the approach demonstrates sufficient accuracy for initial population screening and has strong potential for integration into community health monitoring systems in superaged societies.

## Abbreviations

AUC: area under the receiver operating characteristic curve
COG: center of gravity
GRF: ground reaction force
ICC: intraclass correlation coefficient
SMI: skeletal muscle index
TRIPOD: Transparent Reporting of a Multivariable Prediction Model for Individual Prognosis or Diagnosis

## References

[1] Partridge L, Deelen J, Slagboom PE (2018). Facing up to the global challenges of ageing. Nature.

[2] (2022). Annual Health, Labour and Welfare Report 2022. https://www.mhlw.go.jp/english/wp/wp-hw15/index.html.

[3] Maguire PA, Taylor IC, Stout RW (1986). Elderly patients in acute medical wards: factors predicting length of stay in hospital. Br Med J (Clin Res Ed).

[4] Launay CP, Kabeshova A, Lanoé A, Chabot J, Levinoff EJ, Beauchet O (2018). Age effect on the prediction of risk of prolonged length hospital stay in older patients visiting the emergency department: results from a large prospective geriatric cohort study. BMC Geriatr.

[5] Cunha AIL, Veronese N, de Melo Borges S, Ricci NA (2019). Frailty as a predictor of adverse outcomes in hospitalized older adults: a systematic review and meta-analysis. Ageing Res Rev.

[6] Howlett SE, Rutenberg AD, Rockwood K (2021). The degree of frailty as a translational measure of health in aging. Nat Aging.

[7] Cruz-Jentoft AJ, Baeyens JP, Bauer JM (2010). Sarcopenia: European consensus on definition and diagnosis: report of the European Working Group on Sarcopenia in Older People. Age Ageing.

[8] Fiatarone MA, Marks EC, Ryan ND, Meredith CN, Lipsitz LA, Evans WJ (1990). High-intensity strength training in nonagenarians. Effects on skeletal muscle. JAMA.

[9] Dent E, Martin FC, Bergman H, Woo J, Romero-Ortuno R, Walston JD (2019). Management of frailty: opportunities, challenges, and future directions. Lancet.

[10] Trevisan C, Vetrano DL, Calvani R, Picca A, Welmer AK (2022). Twelve-year sarcopenia trajectories in older adults: results from a population-based study. J Cachexia Sarcopenia Muscle.

[11] Alcazar J, Losa-Reyna J, Rodriguez-Lopez C (2018). The sit-to-stand muscle power test: an easy, inexpensive and portable procedure to assess muscle power in older people. Exp Gerontol.

[12] Park C, Sharafkhaneh A, Bryant MS, Nguyen C, Torres I, Najafi B (2021). Toward remote assessment of physical frailty using sensor-based sit-to-stand test. J Surg Res.

[13] Cruz-Jentoft AJ, Bahat G, Bauer J (2019). Sarcopenia: revised European consensus on definition and diagnosis. Age Ageing.

[14] Fried LP, Tangen CM, Walston J (2001). Frailty in older adults: evidence for a phenotype. J Gerontol A Biol Sci Med Sci.

[15] Obuchi SP, Tsuchiya S, Kawai H (2018). Test-retest reliability of daily life gait speed as measured by smartphone global positioning system. Gait Posture.

[16] Obuchi SP, Kawai H, Murakawa K (2020). Reference value on daily living walking parameters among Japanese adults. Geriatr Gerontol Int.

[17] Lusardi MM, Fritz S, Middleton A (2017). Determining risk of falls in community dwelling older adults: a systematic review and meta-analysis using posttest probability. J Geriatr Phys Ther.

[18] Makizako H, Shimada H, Doi T (2017). Predictive cutoff values of the five-times sit-to-stand test and the timed "up & go" test for disability incidence in older people dwelling in the community. Phys Ther.

[19] Meulemans L, Alcazar J, Alegre LM (2023). Sensor- and equation-based sit-to-stand power: the effect of age and functional limitations. Exp Gerontol.

[20] Millor N, Lecumberri P, Gomez M, Martinez-Ramirez A, Izquierdo M (2014). Kinematic parameters to evaluate functional performance of sit-to-stand and stand-to-sit transitions using motion sensor devices: a systematic review. IEEE Trans Neural Syst Rehabil Eng.

[21] Regterschot GRH, Zhang W, Baldus H, Stevens M, Zijlstra W (2014). Test-retest reliability of sensor-based sit-to-stand measures in young and older adults. Gait Posture.

[22] Kera T, Kawai H, Takahashi J (2022). Development of a screening formula for sarcopenia using ground reaction force during sit-to-stand motion. Gait Posture.

[23] Kera T, Kawai H, Takahashi J (2020). Association between ground reaction force in sit-to-stand motion and falls in community-dwelling older Japanese individuals. Arch Gerontol Geriatr.

[24] Ngoh KJH, Gouwanda D, Gopalai AA, Chong YZ (2018). Estimation of vertical ground reaction force during running using neural network model and uniaxial accelerometer. J Biomech.

[25] Pouliot-Laforte A, Veilleux LN, Rauch F, Lemay M (2014). Validity of an accelerometer as a vertical ground reaction force measuring device in healthy children and adolescents and in children and adolescents with osteogenesis imperfecta type I. J Musculoskelet Neuronal Interact.

[26] Wundersitz DWT, Netto KJ, Aisbett B, Gastin PB (2013). Validity of an upper-body-mounted accelerometer to measure peak vertical and resultant force during running and change-of-direction tasks. Sports Biomech.

[27] Straczkiewicz M, James P, Onnela JP (2021). A systematic review of smartphone-based human activity recognition methods for health research. NPJ Digit Med.

[28] Nishiguchi S, Yamada M, Nagai K (2012). Reliability and validity of gait analysis by android-based smartphone. Telemed J E Health.

[29] Grouios G, Ziagkas E, Loukovitis A, Chatzinikolaou K, Koidou E (2022). Accelerometers in our pocket: does smartphone accelerometer technology provide accurate data?. Sensors (Basel).

[30] Kawai H, Ejiri M, Ito K (2023). Social interaction trajectories and all-cause mortality in older adults: the Otassha study. Front Public Health.

[31] Achamrah N, Colange G, Delay J (2018). Comparison of body composition assessment by DXA and BIA according to the body mass index: a retrospective study on 3655 measures. PLoS One.

[32] Chen LK, Woo J, Assantachai P (2020). Asian Working Group for Sarcopenia: 2019 consensus update on sarcopenia diagnosis and treatment. J Am Med Dir Assoc.

[33] Satake S, Arai H (2020). The revised Japanese version of the Cardiovascular Health Study criteria (revised J-CHS criteria). Geriatr Gerontol Int.

[34] Kawai H, Obuchi S, Ejiri M, Ito K (2023). Association between daily life walking speed and frailty measured by a smartphone application: a cross-sectional study. BMJ Open.

[35] Jiménez-Zazo F, Navarrete-Villanueva D, Gómez-Cabello A (2022). Psychosocial factors related to physical activity in frail and prefrail elderly people. BMC Geriatr.

[36] Avgerinou C, Gardner B, Kharicha K (2019). Health promotion for mild frailty based on behaviour change: perceptions of older people and service providers. Health Soc Care Community.

[37] Antonovsky A (1979). Health, Stress, and Coping.

